# Joint frequency space design approach for efficient planar frequency diverse arrays

**DOI:** 10.1038/s41598-023-39024-6

**Published:** 2023-07-23

**Authors:** Maryam Hasheminasab, A. Cheldavi, Ahmed A. Kishk

**Affiliations:** 1 Electrical Engineering Department, Iran University of Science and Technology, Tehran, 1684613114 Iran; 2 Engineering, Computer Science and Visual Arts Integrated Complex, Concordia University, Montreal, H3G 1M8 Canada

**Keywords:** Energy science and technology, Engineering, Mathematics and computing

## Abstract

Antenna arrays offer advantages in terms of spatial diversity, allowing for control over pattern specifications in space. The incorporation of frequency diversity in arrays presents an opportunity to manipulate beams in the Space–Time domain. Unlike conventional arrays, Frequency Diverse Arrays (FDA) with added frequency diversity exhibit time-variant and range-dependent patterns. These time variations impact both steering and auto-scanning applications. The array factor is influenced by the coherent interplay between frequency and spatial distributions of elements, thereby correlating the spatial and temporal behavior of the FDA’s pattern. To address this space-frequency coherency, an adjoint spatial-frequency design algorithm is the most effective approach to controlling the array's spatial and temporal behavior. Given the complexity of the array factor formulations in FDAs, elements' frequency and spatial distribution have traditionally been designed separately. However, this study proposes an algorithm that simultaneously allocates the location and frequency of elements to achieve a desired pattern. Symmetrical FDA is initially designed using a straightforward formulation of the array factor obtained through symmetry, ensuring a stable and periodic scanning beam. Subsequently, two important design parameters and several crucial design criteria for scanning applications are suggested by analyzing the formulations. These parameters form the basis of a designing algorithm that enables the simultaneous design of element location and frequency in the space-frequency plan, thus meeting the temporal and spatial requirements of the pattern. To demonstrate the efficacy of the proposed algorithm, two different planar arrays are designed, and their results are compared with those of other planar configurations. This study lays the foundation for a novel approach to designing Frequency Diverse Arrays (FDAs), opening up new possibilities in array design.

## Introduction

In a Frequency Diverse Array (FDA), adjacent elements are deliberately given a slight frequency offset from the operating frequency. This unique characteristic sets FDAs apart from conventional arrays, as this incremental frequency offset leads to changes in the array pattern over time and range. However, designing FDAs to achieve the desired pattern with optimal frequency and location distributions for the elements remains a significant challenge. Previous studies on FDA have primarily focused on investigating the characteristics of linear and planar arrays for beam steering applications^1–3^. An “optimization” or “comparing multiple configurations” approach has been commonly employed to achieve optimal pattern performance. This involves simulating and comparing different frequency and amplitude distributions for planar and linear arrays^4–6^. The initial FDA designs were linear in geometry^7^, but subsequent studies expanded to planar configurations^8^. Planar configurations appeal to designers due to their higher gain and directivity than linear configurations^9^. Critical investigations of planar FDAs can be found in^10,11^. In^10^, a rectangular array with a 2-D frequency increment was proposed and analyzed, while in^11^, frequency diversity was applied in a single dimension. Both studies employed uniformly distributed array geometry and frequency offsets. Subsequently, a circular FDA geometry was proposed in^3^, where a ring array with uniform frequency offsets was simulated. Other frequency distributions, such as tangent hyperbolic^12^ and cosine functions^4,13^ have also been explored. Most of these studies have focused on beam steering applications in radar systems. However, using FDA for beam steering poses a critical challenge: pattern fluctuations and stability over time.

Various techniques have been proposed to address pattern stability and fix the pattern in a specific angle, range, and time for pulsed signals. One approach involved time modulation of the frequency distribution of the elements^14^, but this method was later proven invalid^15^. Other techniques utilized mixers and filters^16^. In^17^, a hemispherical conformal FDA structure with an optimized static nonlinear frequency offset was designed to minimize pattern fluctuations^18^. Suggestions such as using sinusoidal element spacing with sinusoidal frequency distribution for a linear array were presented in^19^. Also, articles proposed baseband modulation, multibeam index^20^, and baseband modeling of FDAs^21^. While these techniques are valuable, their complexity and reliance on complicated algorithms limit their practical applicability. As pattern fluctuation arises from the frequency and spatial diversity of the elements, recent research has proposed investigating different spatial configurations to identify the optimal array setup^22^. Another significant application of the FDA is in scanning arrays. The frequency differences between elements in an FDA result in periodic phase differences, enabling the main beam direction to scan the space continuously^23^. This property eliminates the need for phase shifters typically used in conventional arrays, which have power handling and bandwidth limitations, in addition to the RF chain losses. In FDA, the scan rate of the pattern is determined by the frequency and spatial distribution of the elements. It represents the rate at which the scanning pattern repeats during a specific time window. Unlike conventional phased arrays, the scanning FDA exhibits varying angle-changing rates for different angles in the scanning cycle. The scan rate of each Array Factor (AF) term depends on the frequency and location offsets of the corresponding element from the reference element in the array, and these values may differ among AF terms. This non-periodicity in the scan rate and angle-changing rate of elements is reflected in the range-angle pattern. As a result, for the same scanning angle, the gain and SLL vary in each scanning cycle. This unpredictability in the pattern of the FDA limits its benefits in scanning applications. The angle-changing rate is defined as $\frac{{\partial \theta_{\max } \left( {\theta ,\varphi } \right)}}{\partial t}$, generally depends on the elevation and azimuth angle of the main beam. While the auto-scan capability is advantageous in FDAs, ensuring the array’s time behavior stability is crucial before utilizing this capability. Achieving practical applications of FDA techniques requires simultaneous control of the time and spatial behavior of the array.

In this study, we propose an adjoint space-frequency algorithm to fully control the time and spatial behavior of the main beam in FDAs. To develop the algorithm, we first derive a straightforward analytical formula for the Array Factor (AF) by utilizing symmetry definitions for the array configuration. This symmetry ensures the desired symmetrical scanning pattern. Next, we employ the derived formula to calculate and unify each term’s scan periodicity and angle-changing rate in the AF. Using these two parameters as design parameters for the FDA, we propose an adjoint design algorithm and flowchart to allocate the frequency and spatial distribution of the FDA. We apply the proposed algorithm to design a discular FDA that exhibits stable scanning beam characteristics. To validate the algorithm’s superiority, we redesign and simulate multiple commonly used configurations, comparing their results with those of the designed array. To comprehensively compare beam specifications, we propose three meaningful pattern planes in the time-spatial domain and utilize them to evaluate the array performances. Additionally, we introduce a space-frequency diagram for analyzing FDA distributions and compare the geometries of the best and worst simulated arrays in the space-frequency diagram.

Section “Theoretical analysis and formulation” applies mathematical simplification to derive a general straightforward formula for the AF. Section “Designing algorithm for planar frequency diverse scanning array” presents the design algorithm and introduces critical design parameters of the FDA. Using the derived formulation and proposed algorithm, we design a discular array. In Sections “Redesigning and simulation of some typical geometries” and “Comparison of the array’s performances”, we redesign and simulate other commonly used FDA configurations to evaluate their performance and compare them with the designed array. These redesigns are necessary as comprehensive data for comparing array performance were unavailable in the literature. Finally, Section “Conclusion” concludes the study and discusses potential future research directions.

## Theoretical analysis and formulation

This study focuses on scanning applications of Frequency Diverse Arrays (FDA), where a symmetrical pattern in space is often desired. To ensure the symmetrical scanning pattern, we introduce frequency and spatial symmetry criteria, which are employed to derive a straightforward equation for the array factor of the FDA. It is important to note that while the primary aim of this paper is scanning applications, the derived formulation can also be applied to other applications or geometries that meet the symmetry criteria. For a one-directional symmetrical scanning pattern along the x-axis, the FDA requires specific symmetries in the frequency and spatial distributions. Even symmetry along the X- and Y-axis is considered for the spatial distribution. This means that the placement of the antennas exhibits symmetry when reflected along both axes. On the other hand, for the frequency distribution, an incremental frequency distribution along the X-axis with odd symmetry and even symmetry along the Y-axis is defined. These symmetries will be further elaborated in the relevant equations in subsequent sections.

**Figure 1:**
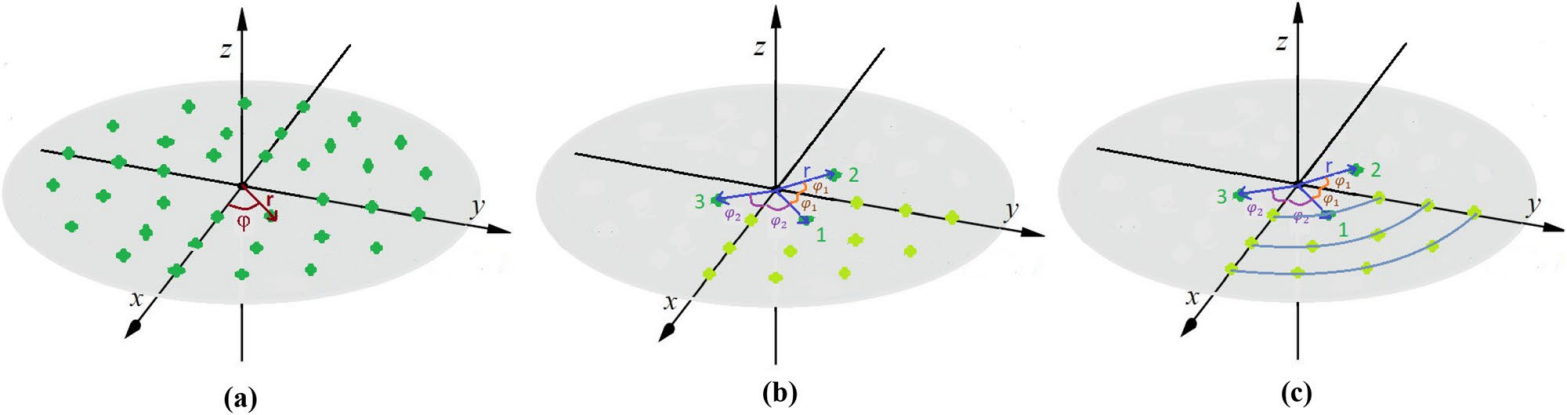
(**a**) General array configuration, (**b**) one-quarter of the array elements with x- and y-axis symmetry, (**c**) Rings of the discular array.

The planar arrays are conceptualized as a combination of multiple distinct rings in the array plane with arbitrary shapes to form the array factor. Figure 1a provides a visual representation of a general array configuration in cylindrical coordinates, where each element's location can be described using cylindrical coordinates (r, φ) which is illustrated in Fig. 1b. For example, Fig. 1c illustrates a planar array composed of circular rings called a Discular Array. In general, a planar array can consist of M rings, with each ring containing ${N}^{i}$ elements (i ranging from 1 to M). Throughout the subsequent sections, in addition to the Discular array, the rectangular and hexagonal array configurations with rectangle and hexagon rings, respectively, are also investigated.

### Array factor derivation for FDA

To calculate the Array Factor (AF), we need to sum up the contributions of each element to the overall pattern. The formulation for the frequency diverse array’s AF in cylindrical coordinate system, considering an array with *M* rings and *N*^*i*^ elements in the *i*^*th*^ ring can be expressed as Eq. (1).

1 $$ AF(r,\theta ,\;\varphi ;\;t) = \sum\limits_{i = 0}^{M} {\sum\limits_{n = 1}^{{N^{i} }} {A_{n} e^{{j\omega_{n}^{i} \frac{{\rho^{i} sin\theta \;\cos (\varphi - \varphi_{n}^{i} )}}{c}}} e^{{j\delta \omega_{n}^{i} (t - \frac{{R_{0} }}{c})}} } } $$

In this formulation far field assumption has been considered ($\left|{{\varvec{R}}}_{0}\right|\gg \left|{{\varvec{r}}}_{n}^{i}\right|)$ and it has been normalized by the term $exp(j\omega_{0} (t - \frac{{R_{0} }}{c}))/R_{0}$. The *i* = 0 index represents the central element, and $\rho^{i}$ and $\varphi_{n}^{i}$ define the *n*^th^ element position in the *i*^th^ ring and the frequency of each element is obtained from $\omega_{n}^{i} = \omega_{0} + \delta \omega_{n}^{i}$ . For deriving Eq. (1) it has been assumed that the frequency difference between the elements is much smaller than the central frequency, we can write; $\delta {\omega }_{n}^{i}\ll {\omega }_{0}$. Equation (1) can also be applied to conventional arrays, except that the frequency-dependent term becomes zero. To enhance the formula's simplicity, we consolidate all the fixed parameters of the array into a single symbol. Furthermore, we introduce the variables $\tau = t - \frac{{R_{0} }}{c}$ and $\gamma_{n}^{i} (\theta ) = \omega_{n}^{i} \frac{{\rho^{i} }}{c}\sin \theta$ represent the exponent's time and frequency dependencies, respectively. These notations can express the array factor (AF) as follows

2 $$ AF(r,\theta ,\;\varphi ,\;t) = \sum\limits_{i = 0}^{M} {\sum\limits_{n = 1}^{{N^{i} }} {A_{n}^{i} e^{{j\gamma_{n}^{i} (\theta )\cos (\varphi - \varphi_{n}^{i} )}} e^{{j\delta \omega_{n}^{i} \tau }} } } $$

The first exponent in the equation represents the spatial behavior, while the second exponent determines the time behavior of the array factor (AF). The term $\gamma_{n}^{i} (\theta )$ demonstrates the coherence between time and spatial behaviors. In conventional arrays, this term can be simplified to $\gamma_{n}^{i} (\theta ) = 2\pi \frac{{\rho^{i} }}{{\lambda_{0} }}\sin \theta$, relying solely on the spatial location of the element. In the case of the FDA, this term takes the form $\gamma_{n}^{i} (\theta ) = \left( {\omega_{0} + \delta \omega_{n}^{i} } \right)\frac{{\rho^{i} }}{c}\sin \theta = 2\pi \left( {\frac{{\rho^{i} }}{{\lambda_{0} }}} \right)\left( {1 + \frac{{\delta \omega_{n}^{i} }}{{\omega_{0} }}} \right)\sin \theta$. The second parentheses represent the modulation of the radii. By comparing these two terms in FDA, we can define the effective radius of each element as, $\rho_{eff} = \rho^{i} \left( {1 + \frac{{\delta \omega_{n}^{i} }}{{\omega_{0} }}} \right)$. The effective radius of elements in the FDA is influenced by the frequency offset of each element. However, if $\delta \omega_{n}^{i} < < \omega_{0}$, the effective and the actual radii of the elements converge. This concept provides a clearer explanation for the variation in the spatial behavior of the array pattern by changing the frequency distribution of the elements. In Eq. (2), the number of summations is equal to the number of array elements. Although, by applying the defined odd and even symmetries around the x and y-axis, the number of independent terms is reduced. To maintain x-axis and y-axis symmetry in the element placement, the number of elements in each ring should be even. If the array elements are uniformly distributed along the ring, the angle of each element can be calculated as:

3 $$ \varphi_{n}^{i} = \frac{2\pi }{{N^{i} }}(n - 1) + \varphi_{0} \quad \quad \quad n = 1,2,...N^{i} ,\left( {N^{i} :even} \right) $$

where $\varphi_{0}^{i}$ determines the location phase of the first element in the *i*th ring of the array while $\varphi_{0}$ denotes the rotation angle $\varphi_{0}^{i}$ from the x-axis. Assuming the frequency distribution of the ring to be oddly symmetric along the x-axis and evenly symmetric along the y-axis, we can express Eq. (4a) for the frequency distribution of the elements. The notation $N_{h}^{i}$ in Eq. (4a) is defined as half of the total number of elements in the $i^{th}$ ring, ($N_{h}^{i}  = N^{i} /2$). Additionally, in Eq. (4b) the excitation amplitudes are defined to be evenly symmetric along the x-axis and y-axis.

4a $$ \delta \omega_{n}^{i} = \left\{ {\begin{array}{*{20}l} { - \delta \omega_{{N_{h}^{i} - n + j}}^{i} } \hfill & {n \le N_{h}^{i} } \hfill \\ {\delta \omega_{{N^{i} - n + j}}^{i} } \hfill & {n > N_{h}^{i} } \hfill \\ \end{array} } \right.\quad when\;j = \left\{ {\begin{array}{*{20}l} 2 \hfill & {elements\;on\;x - axis} \hfill \\ 1 \hfill & {no\;elements\;on\;x - axis} \hfill \\ \end{array} } \right. $$

4b $$ A_{n}^{i} = \left\{ {\begin{array}{*{20}l} {A_{{N_{h}^{i} - n + j}}^{i} } \hfill & {n \le N_{h}^{i} } \hfill \\ {A_{{N^{i} - n + j}}^{i} } \hfill & {n > N_{h}^{i} } \hfill \\ \end{array} } \right.\quad when\;j = \left\{ {\begin{array}{*{20}l} 2 \hfill & {elements\;on\;x - axis} \hfill \\ 1 \hfill & {no\;elements\;on\;x - axis} \hfill \\ \end{array} } \right. $$

By utilizing the suggested symmetries for the frequency, amplitudes, and element locations, we can derive the AF formulation by considering only the parameters of the elements located in one-quarter of the array plane. This reduction in the required information simplifies the calculation process and facilitates the analysis of the array's behavior. It should be noticed that many FDA geometries designed for both steering and scanning application could have the defined symmetry^7,22–26^. The linear or sinusoidal frequency distributions can also fulfill the symmetry rules described earlier. The relationship between the linear and cosine distributions, which is used in the following sections of the manuscript, can be expressed as follows:

5 $$ \delta \omega_{n}^{i} = \omega_{b} \left( {1 - \frac{2}{\pi }\varphi_{n}^{i} } \right)\quad n \le N_{h}^{i} \;\left( {{\text{Linear}}} \right) $$

6 $$ \delta \omega_{n}^{i} = \omega_{b} \cos \varphi_{n}^{i} \quad n \le N_{h}^{i} \;\left( {{\text{Cosine}}} \right) $$

In (5) and (6), ${\omega }_{b}$ is the fundamental beat frequency of the array. By collecting symmetric elements along the x-axis, the AF for the elements above the x-axis could be obtained:

7 $$ AF_{U} (r,\theta ,\;\varphi ,\;t) = \sum\limits_{i = 0}^{M} {\left[ {\left\{ {2\sum\limits_{n = 1}^{{N_{q}^{i} + j - 1 - CF}} {A_{n}^{i} e^{{j\psi_{sn}^{i} (\theta ,\varphi )}} \cos (\psi_{cn}^{i} (\theta ,\varphi ) + \delta \omega_{n}^{i} \tau )} } \right\} + m_{y}^{i} A_{{N_{q}^{i} + j - CF}}^{i} e^{{j\psi_{{s(N_{q}^{i} + j - CF)}}^{i} (\theta ,\varphi )}} } \right]} $$

$$ m_{{\left\{ {\begin{array}{*{20}c} x \\ y \\ \end{array} } \right\}}}^{i} = \left\{ {\begin{array}{*{20}l} 0 \hfill & {no\;elements\;on\;\left\{ {\begin{array}{*{20}c} x \\ y \\ \end{array} } \right\} - axis} \hfill \\ 1 \hfill & {elements\;on\;\left\{ {\begin{array}{*{20}c} x \\ y \\ \end{array} } \right\} - axis} \hfill \\ \end{array} } \right. $$

where $N_{q}^{i}$ is the number of elements in one-quarter of the array plan and defined as $N_{q}^{i} = N_{h}^{i} /2 = N^{i} /4$. CF is the adjusting factor that represents the location of the first element of the ring [Appendix A]. In Eq. (7) ${\psi }_{cn}^{i}(\theta ,\hspace{0.33em}\varphi )={\gamma }^{i}(\theta )\mathit{cos}\phi \mathit{cos}{\varphi }_{n}^{i}$ and ${\psi }_{sn}^{i}(\theta ,\hspace{0.33em}\varphi )={\gamma }^{i}(\theta )\mathit{sin}\phi \mathit{sin}{\varphi }_{n}^{i}$. In case where the rotation angle of the array is set to zero, $\varphi_{0}^{i} = 0$, the AF of the elements located below the x-axis is obtained from:

8 $$ AF_{L} (r,\theta ,\;\varphi ;\;t) = \sum\limits_{i = 0}^{M} {\left[ {\left\{ {2\sum\limits_{{n = N_{h}^{i} + j - CF + m_{y}^{i} }}^{{N^{i} }} {A_{n}^{i} e^{{j\psi_{sn}^{i} (\theta ,\varphi )}} \cos (\psi_{cn}^{i} (\theta ,\varphi ) + \delta \omega_{n}^{i} \tau )} } \right\} + m_{y}^{i} A_{{N_{h}^{i} + N_{q}^{i} + j - CF}}^{i} e^{{j\psi_{sn}^{i} (\theta ,\varphi )}} } \right]} $$

By summing up the terms with the same frequency offsets in (7) and (8) and using sinusoidal simplifications, we have:

9a $$ AF(r,\theta ,\;\varphi ;\;t) = \sum\limits_{i = 0}^{M} {2m_{y}^{i} A_{{N_{q}^{i} + j - CF}}^{i} \cos (\psi_{{sN_{q}^{i} + j - CF}}^{i} (\theta ,\varphi ))} + 2m_{x}^{i} A_{1}^{i} \cos (\psi_{c1}^{i} (\theta ,\varphi ) + \delta \omega_{1}^{i} \tau ) + 4\left\{ {\sum\limits_{n = j}^{{N_{q}^{i} + j - 1 - CF}} {A_{n}^{i} \cos (\psi_{sn}^{i} (\theta ,\varphi ))\cos (\psi_{cn}^{i} (\theta ,\varphi ) + \delta \omega_{n}^{i} \tau )} } \right\} $$

9b $$ AF(r,\theta ,\;\varphi ;\;t) = \sum\limits_{i = 0}^{M} {a_{i} \sum\limits_{n = 1}^{{N_{q}^{i} + \{ m_{y}^{i} or\;m_{y}^{i} \} }} {A_{n}^{i} \cos (\psi_{sn}^{i} (\theta ,\varphi ))\cos (\psi_{cn}^{i} (\theta ,\varphi ) + \delta \omega_{n}^{i} \tau )} } $$

where $a_{n} = 2$ for $\phi_{1}^{i} = 0$, $\phi_{{N_{q}^{i} + \{ m_{y}^{i} or\;m_{y}^{i} \} }}^{i} = \pi /2$, and $a_{n} = 4$ for others $\phi_{n}^{i}$. By extracting $\psi_{sn}^{i} (\theta ,\varphi )$ and $\psi_{cn}^{i} (\theta ,\varphi )$, Eq. (9b) can be simplified into:

10 $$ AF(r,\theta ,\;\varphi ;\;t) = \sum\limits_{i = 0}^{M} {a_{i} \sum\limits_{n = 1}^{{N_{q}^{i} + \{ m_{y}^{i} or\;m_{y}^{i} \} }} {A_{n}^{i} \cos (\gamma_{n}^{i} (\theta ) + \delta \omega_{n}^{i} \tau )\cos (\varphi )\cos (\varphi_{n}^{i} )} } $$

Equation (10) has $N_{q}^{i} = N^{i} /4$ separate terms, and each term is obtained based on the multiplication of three cosine functions. For the conventional array ${\omega }_{n}=0$, and the formula converges to:

11 $$ AF(r,\theta ,\;\varphi ;\;t) = \sum\limits_{i = 0}^{M} {a_{i} \sum\limits_{n = 1}^{{N_{q}^{i} + \{ m_{y}^{i} or\;m_{y}^{i} \} }} {A_{n}^{i} } } \cos \left( {\omega_{0} \frac{{\rho_{n}^{i} }}{c}\sin (\theta )(\cos (\varphi )\cos (\varphi_{n}^{i} )} \right) $$

While this formulation can simplify the array factor (AF) for conventional arrays, its advantages become even more significant in the design of Frequency Diverse Arrays (FDA). In the upcoming section, we utilize this formulation to calculate the scan period and angle-changing rate of arrays. Furthermore, we design a discular array that exhibits a stable scanning beam.

## Designing algorithm for planar frequency diverse scanning array

In this section, first, using derived formulations, the time behavior of the pattern is analyzed. Moreover, the time behavior of the pattern is analyzed using the derived formulations. Based on the analysis results and by defining certain constraints for the geometry and frequency distribution, we propose a design algorithm. This algorithm aims to achieve a stable and invariant pattern for all scan angles.

### FDA pattern analysis

In the AF equation, the argument of the second cosine function in (9b) determines the time behavior of the pattern. By substituting the expanded relation of $\psi_{cn}^{i} (\theta ,\varphi )$, this term can be written as follows:

12 $$ \cos (\psi_{cn}^{i} (\theta ,\varphi ) + \delta \omega_{n}^{i} \tau ) = \cos (\gamma_{n}^{i} (\theta )\cos \phi \cos \phi_{n}^{i} + \delta \omega_{n}^{i} \tau ) $$

Both the argument of the cosine function and its first derivative are determinative in the time behavior of the pattern. The scan rate can be calculated from the arguments of each term in the AF, while their first derivatives indicate the rate of change of the main beam during the scanning process. The pattern scanning period can be calculated from the scan rate of the array; which is $T_{t} = 2\pi /\delta \omega_{n}^{i}$ for each term. The angle-changing rate of each term can be obtained as follows.

13 $$ \left. {\frac{{d\theta_{\max } }}{dt}} \right|_{n}^{i} = \frac{{\delta \omega_{n}^{i} }}{{\frac{{\omega_{0} }}{c}\rho_{n}^{i} \cdot \cos (\varphi_{\max } ) \cdot \cos (\varphi_{n}^{i} )}} \cdot \frac{1}{{\cos (\theta_{\max } )}} $$

Equation (13) indicates that the angle-changing rate varies for different main beam directions (${\theta }_{max},{\varphi }_{max})$. This variation implies that the maximum pattern of each element rotates at different speeds at different angles, resulting in variations in gain and beamwidth with changing angles and time. Moreover, this leads to fluctuations in the pattern over scanning rounds, as the scan periods of the AF terms could not be unified like the traditional arrays due to the inherent frequency diversity of the array.

While a unique scan rate or angle-changing rate can generate a stable pattern, it is important to note that there may exist other frequency and spatial distributions that can also achieve a stable pattern. Therefore, finding the optimum frequency and spatial distributions that satisfy the stability requirements is a crucial aspect of FDA design, and it is the main objective of the proposed algorithm. The algorithm aims to identify and optimize these distributions to achieve a stable and predictable scanning pattern in FDA applications. The angle-changing rate of each term of the AF depends on $\delta {\omega }_{n}^{i}$, ${\rho }_{n}^{i}$, and ${\varphi }_{n}^{i}$ simultaneously. There may be multiple combinations of these parameters that fulfill the spatial or temporal requirements of the pattern for specific steering angles. However, it does not guarantee that the same combinations will work effectively for other angles. Usually, by choosing the parameters for the best spatial performance, the pattern periodicity degrades, and vice versa, and there is often a trade-off between spatial performance and pattern periodicity. However, the time behavior of the array can be controlled by adjusting and designing the time parameters of the array. This technique mitigates the level of SLL that arises due to the asynchronous timing of the AF terms. By employing Eq. (13) and ensuring a consistent angle-changing rate for all the terms in the AF, a relationship can be established between the $\delta \omega_{n}^{i}$, $\rho_{n}^{i}$, and $\varphi_{n}^{i}$ of the array elements and those of the reference element. In this context, the reference element is typically selected as the first element of the first ring.

14 $$ \frac{{\delta \omega_{n}^{i} }}{{\frac{{\omega_{0} }}{c}\rho_{n}^{i} \cdot \cos (\varphi_{n}^{i} )}} = \frac{{\delta \omega_{1}^{1} }}{{\frac{{\omega_{0} }}{c}\rho_{1}^{1} \cdot \cos (\varphi_{1}^{1} )}} $$

By equating the angle-changing rates of all AF terms, we can derive the necessary relationships between the parameters. This allows for the determination of the spatial and frequency distributions of the elements with the reference element. The derived equations provide valuable insights into the design process and enable the optimization of the FDA’s performance and stability. Since Eq. (14) represents the only equation among the three unknowns ($\delta \omega_{n}^{i}$, $\varphi_{n}^{i}$, $\rho_{n}^{i}$), the equation system does not have a unique solution. However, by rearranging Eq. (14), we can establish a relationship between the frequency offset of the nth term in the ith ring and the frequency offset of the reference element.

15 $$ \delta \omega_{n}^{i} = \delta \omega_{1}^{1} \cdot \left( {\frac{{\rho_{n}^{i} }}{{\rho_{1}^{1} }}} \right) \cdot \cos (\varphi_{n}^{i} ) $$

For circular rings, the cosine frequency distribution satisfies Eq. (14), as also investigated in^6^. In the case of a discular array with N circular rings, the frequency offsets can be obtained as $\delta \omega_{n}^{i} = k.i.\delta \omega_{1}^{1} .\cos (\varphi_{n}^{i} )$, where ‘k’ represents the division factor of the ring's radii. The maximum frequency offset of each ring is also determined by k. The scan periodicity of each term of the AF for such a discular array is given by, $T_{t} = \frac{2\pi }{{\delta \omega_{n}^{i} }} = \frac{K}{{\cos (\varphi_{n}^{i} )}}$ which can be expressed in the form of the co-secant function. The overall scan periodicity of the pattern is determined by calculating the Least Common Multiple (LCM) of the scan periods of all terms. In the case of a cosine frequency distribution, the scattered values of ${T}_{t}$ can potentially yield a high LCM value. This non-uniform distribution of scan periods leads to a non-periodic time behavior of the array and can result in increased Side Lobe Levels (SLLs) in the pattern. In the next section, the design algorithm for achieving optimum time-spatial behavior is presented.

### Design guide for the scanning FDA

In FDAs, due to the time dependency of the pattern, in addition to the Elevation-Azimuth plane (constant time), which is used to simulate and analyze patterns in conventional arrays, two additional planes need to be defined. These planes are the Elevation-time plane (constant azimuth angle and sweeping time) and the Elevation-Range plane (constant azimuth angle and sweeping time). Also, for the design of FDAs, two additional time-related parameters are introduced alongside the spatial distribution. The spatial distribution, scan rate, and angle-changing rate are three interrelated parameters that collectively contribute to the specifications of the array pattern. Based on the analytical results obtained in the previous section, three constraints must be satisfied to achieve stable time behavior and minimize sidelobe levels (SLLs). The three parameters of the array ($\delta \omega_{n}^{i}$, $\varphi_{n}^{i}$, and $\rho_{n}^{i}$) should be designed in the way that:

(I) The angle-changing rate is the same for all the AF terms,
(II) The maximum distance between the elements must be kept under $${\raise0.7ex\hbox{${\lambda_{0} }$} \!\mathord{\left/ {\vphantom {{\lambda_{0} } 2}}\right.\kern-0pt} \!\lower0.7ex\hbox{$2$}}$$.
(III) A harmonic scan period for the AF’s terms.

The design algorithm for FDAs follows a systematic approach to achieve the desired array performance. Here is a breakdown of the algorithm:

1) Determine the number of elements based on the required gain considering a uniform amplitude distribution.

2) Calculate the number of rectangular rings based on the total number of elements.

- The rectangular lattice is chosen for its optimal performance in one-dimensional scanning^11^. It will be converted to circular shapes in the subsequent steps of the design algorithm.

3) Calculate the minimum frequency offset ($\omega_{b}$) of the elements based on the minimum required scan rate.

- This step ensures that the array can achieve the desired scan rate.

**Figure 2:**
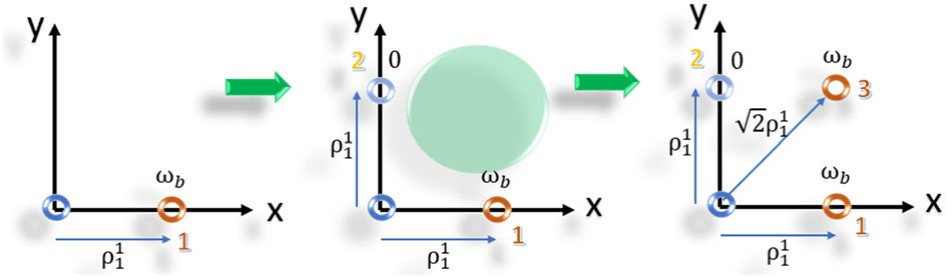
Illustrative design procedure of designing the first ring in an optimal array.

The design algorithm continues with the allocation of elements in the rings. The following steps outline the necessary procedures for this task:

4) Allocate the first ring elements (Fig. 2):

4.1) The first (reference) element is placed at the center of the cylindrical coordinate as the reference element ($\delta \omega_{0}^{0} = 0$, $\varphi_{0}^{0} = 0$, $\rho_{0}^{0} = 0$).

4.2) The first element of ring one is placed along the x-axis with parameters ($$\delta \omega_{1}^{1} = \omega_{b}$$, $$\varphi_{1}^{1} = 0$$, $$\rho_{1}^{1} = {\raise0.7ex\hbox{${\lambda_{0} }$} \!\mathord{\left/ {\vphantom {{\lambda_{0} } 2}}\right.\kern-0pt} \!\lower0.7ex\hbox{$2$}}$$) (Fig. 2a). (Considering the rotation angle of the rings to be zero), the frequency offset of this element is calculated by $$\delta \omega_{1}^{1} = \omega_{b} = scan\;rate$$.

4.3) The second element is placed on the y-axis. Since the array is designed to scan the space in a single direction along the x-axis, the frequency offset of the array elements located along the y-axis is set to zero. For the second element, we have ($$\delta \omega_{2}^{1} = 0$$, $$\varphi_{2}^{1} = {\raise0.7ex\hbox{$\pi $} \!\mathord{\left/ {\vphantom {\pi 2}}\right.\kern-0pt} \!\lower0.7ex\hbox{$2$}}$$, and $$\rho_{2}^{1} = {\raise0.7ex\hbox{${\lambda_{0} }$} \!\mathord{\left/ {\vphantom {{\lambda_{0} } 2}}\right.\kern-0pt} \!\lower0.7ex\hbox{$2$}}$$) (Fig. 2b).

- Generally, $$\rho_{2}^{1}$$($${d}_{y})$$ can be different from $$\rho_{1}^{1}$$($${d}_{x}$$) but for more simplicity we choose $${d}_{x}={d}_{y}$$.
- The maximum distance between elements 1 and 2 exceeds ${\lambda }_{0}/2$, indicating that an additional element needs to be inserted in between

4.4) Choose the line $\varphi  = \pi /4$ to be the location of the third element.

**Figure 3:**
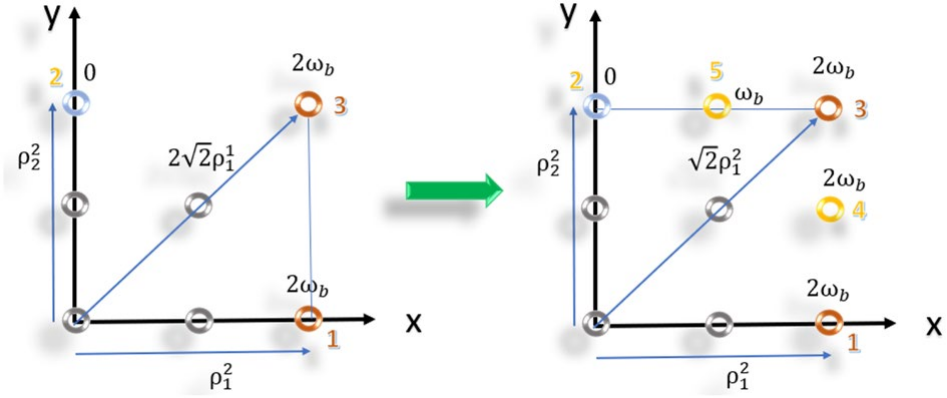
Illustrative design procedure of designing the second ring in the optimal array.

- To preserve symmetry and minimize the maximum distance between elements, the diagonal line is chosen as the placement path.
- The third element introduces a new independent term to the AF. To unify the scan rates of the second and third elements, the same frequency offset (${\omega }_{b}$) is either selected for the third element. By utilizing Eq. (15), the $\rho_{3}^{1}$ can be calculated for the unique angle changing rate.
  16 $$ \rho_{3}^{1} = \frac{{\delta \omega_{3}^{1} }}{{\delta \omega_{1}^{1} }}.\frac{{\rho_{1}^{1} }}{{\cos (\varphi_{3}^{1} )}} = \rho_{1}^{1} .\sqrt 2 $$
- For the first ring, the maximum distance between the elements is < $${\raise0.7ex\hbox{${\lambda_{0} }$} \!\mathord{\left/ {\vphantom {{\lambda_{0} } 2}}\right.\kern-0pt} \!\lower0.7ex\hbox{$2$}}$$, so there is no need to add another element to the ring.

5) Allocate the second ring elements (Fig. 3):

- Follow steps 4.1 to 4.4.
- If constraint II is not satisfied, additional elements are added in between the elements with the maximum distances. The location and frequency of the added elements are determined while considering constraints I and III, as described in Eq. (15).
- The distance between the elements two elements of (1,3) and (2,3) do not satisfy the constant II (Fig. 3). Equation (15) gives the relationship between $\rho_{4}^{2}$ and $\varphi_{1}^{2}$.
  17 $$ \rho_{4}^{2} = \rho_{1}^{2} .\cos (\varphi_{4}^{2} ) $$

Equation (17) describes the geometrical location of all points that satisfy Eq. (15), which involves all the points on the connecting line between elements 1 and 3 in the second ring. To minimize the maximum distance between the elements, the middle of the line is chosen for the element’s location ($$\delta \omega_{4}^{2} = 2\omega_{b}$$, $$\varphi_{4}^{2} = \tan^{ - 1} \left( 2 \right)$$, and $$\rho_{4}^{2} = {\raise0.7ex\hbox{${\lambda_{0} }$} \!\mathord{\left/ {\vphantom {{\lambda_{0} } {\cos \left( {\varphi_{4}^{2} } \right)}}}\right.\kern-0pt} \!\lower0.7ex\hbox{${\cos \left( {\varphi_{4}^{2} } \right)}$}}$$). By applying Eq. (15) for the fifth element, its frequency offset and location are obtained as ($$\delta \omega_{5}^{2} = \omega_{b}$$, $$\varphi_{5}^{2} = \tan^{ - 1} \left( {1/2} \right)$$, and $$\rho_{5}^{2} = {\raise0.7ex\hbox{${\lambda_{0} }$} \!\mathord{\left/ {\vphantom {{\lambda_{0} } {\cos \left( {\varphi_{5}^{2} } \right)}}}\right.\kern-0pt} \!\lower0.7ex\hbox{${\cos \left( {\varphi_{5}^{2} } \right)}$}}$$).

6) Add more rings following the same steps as step 5.

- In general, the location of elements in a designed rectangular lattice can be described by (18).
  18a $$ x_{m} = \left\{ {\begin{array}{*{20}l} {md_{x} } \hfill & {m = 0,1, \ldots ,\frac{{M_{x} - 1}}{2}\quad for{\kern 1pt} \,odd\,M_{x} } \hfill \\ {\left( {2m - 1} \right)\frac{{d_{x} }}{2}} \hfill & {m = 1, \ldots ,\frac{{M_{x} }}{2}\quad for{\kern 1pt} \,even\,M_{x} } \hfill \\ \end{array} } \right. $$
  18b $$ y_{n} = \left\{ {\begin{array}{*{20}l} {nd_{y} } \hfill & {m = 0,1, \ldots ,\frac{{M_{y} - 1}}{2}\quad for{\kern 1pt} \,odd\,M_{y} } \hfill \\ {\left( {2n - 1} \right)\frac{{d_{y} }}{2}} \hfill & {m = 1, \ldots ,\frac{{M_{y} }}{2}\quad for{\kern 1pt} \,even\,M_{y} } \hfill \\ \end{array} } \right. $$

*N*_*y*_ is the number of parallel rows to the x-axis separated by *d*_*y*_, and *M*_*x*_ is the number of parallel columns to the y-axis and separated by *d*_*x*_. In cylindrical coordinates, the element's locations are described by (*ρ*_*m,n*_*, ϕ*_*m,n*_), where $\rho_{m,n} = \sqrt {x_{m}^{2} + y_{n}^{2} }$ and $\varphi_{m,n} = \tan^{ - 1} (y_{n} /x_{n} )$. Elements of each column have a constant frequency offset which is described by the following equation:

19 $$ \omega_{m}^{n} = \left\{ {\begin{array}{*{20}l} {n\omega_{b} } \hfill & {m = 0,1, \ldots ,\frac{{M_{x} - 1}}{2}\quad for{\kern 1pt} \,odd\,M_{x} } \hfill \\ {\left( {2m - 1} \right)\frac{{\omega_{b} }}{2}} \hfill & {m = 1, \ldots ,\frac{{M_{x} }}{2}\quad for{\kern 1pt} \,even\,M_{x} } \hfill \\ \end{array} } \right. $$

The location and frequency offset for other elements in the other quadrants are calculated using the defined symmetry rules. In the designed array to assure symmetry, a rectangular grid is designed, $M_{x} = N_{y} = N_{sq}$, but generally, they can be different. Although, nonsymmetrical x–y geometries lead to a nonsymmetrical pattern in the different azimuth angles.

**Figure 4:**
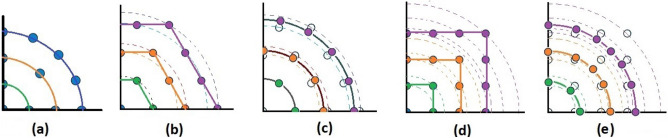
Elements in the first quarter in (**a**) circular, (**b**) hexagonal (**c**) equivalent circular of the hexagonal array, and (**d**) rectangular lattice (**e**) the equivalent circular of the rectangular lattice.

7) Convert the rectangular lattice to a discular array.

- To form the discular array, the elements are relocated to circular rings. During this repositioning process, all the frequency and spatial parameters of the rectangular lattice remain unchanged, except for the radii of the elements in the cylindrical coordinate (Fig. 4e). Although the frequency distribution remains similar to the rectangular lattice, the angle-changing rate of the AF terms in the discular array is different due to the spatial replacement of elements. As a result, Eq. (15) is not satisfied for the discular array. To minimize the perturbations in the angle-changing rate, an error function is defined. This error function is calculated as the sum of the differences between the angle-changing rates of the AF terms in the rectangular and discular arrays. The objective is to find the radii of the equivalent circles in the rings that minimize this error function. The error function represents the deviation between the angle-changing rates of the AF terms in the two array geometries.
  20 $$ F_{e} (\rho_{eq}^{i} )\; = \sum\limits_{i = 0}^{M} {\sum\limits_{n}^{{N_{q}^{i} }} {\left( {\left[ {\frac{d\theta }{{dt}}} \right]_{rect} - \left[ {\frac{d\theta }{{dt}}} \right]_{n(eq)} } \right)} } = K\sum\limits_{i = 0}^{M} {\sum\limits_{n = 1}^{{N_{q}^{i} }} {\frac{{\delta \omega_{1}^{i} }}{{\rho_{n}^{i} }} - \frac{{\delta \omega_{n}^{i} }}{{\rho_{eq}^{i} \cos \varphi_{n}^{i} }}} } = 0 $$
  $$ K = \frac{1}{{\frac{{\omega_{0} }}{c} \cdot \cos (\varphi_{\max } ) \cdot \cos (\theta_{\max } )}} $$

By setting the error function to zero and substituting (15), the equivalent radius of each ring that satisfies (20) is obtained.

21 $$ \rho_{eq}^{i} \; = \frac{1}{{N_{q}^{i} }}\;\sum\limits_{n}^{{N_{q}^{i} }} {\rho_{1}^{i} \csc \left( {\varphi_{n}^{i} } \right)} = \frac{1}{{N_{q}^{i} }}\sum\limits_{n = 1}^{{N_{q}^{i} }} {\rho_{n}^{i} } = ave\left( {\rho_{n}^{i} } \right) $$

Surprisingly, Eq. (21) represents the exact equation for the average radii of the rings (as shown in Appendix B). In the next section, in addition to the designed array, five other commonly used geometries for FDAs in the literature are redesigned and simulated, and their results are compared with those of the designed array results.

## Redesigning and simulation of some typical geometries

To facilitate a comprehensive comparison, multiple commonly used geometries for FDAs have been redesigned and simulated alongside the designed array. These geometries include discular, hexagonal, and rectangular shapes with linear, cosine, and other frequency distributions, which have been previously utilized in various references^2,5,8,9,13,22,24^. The purpose of redesigning these arrays is twofold. Firstly, it allows for the collection of necessary data to compare their radiation patterns, as such information was not readily available in the existing literature. Secondly, it enables a fair comparison by eliminating the influence of the total number of elements in the evaluation process. During the redesigning process, only the array geometries and frequency distributions have been retained, while the number of elements has been adjusted to match the nearest value of the designed array. This ensures a consistent evaluation of the array geometries and frequency distributions while eliminating the impact of the total number of elements. For each selected geometry, an equivalent discular array has also been designed using a similar procedure as the one employed for the designed array. This involves determining the radii of the rings in the equivalent discular array while keeping the other spatial and frequency distributions unchanged. Figure 4 provides a visual representation of the redesigned arrays and their equivalent discular arrays, showcasing the different geometries and their corresponding designs.

On the other hand, to better compare the time and spatial behavior of the FDAs, a Figure of Merit (FoM) is defined based on the side lobe level (SLL) of the pattern in three defined planes. The side lobes in the range-angle and elevation-time planes demonstrate the time periodicity of the array, while the maximum side lobe level in the elevation-azimuth plane over multiple periods represents the spatial behavior of the array. The FoM is calculated as the sum of the maximum SLL in these three planes, divided by the maximum gain of the array.

22 $$ FoM = \sum\limits_{n = 1}^{3} {\frac{{SLL_{Plane - n} }}{\max (AF)}} $$

The first simulated array, A1, is a three-ring discular array with a cosine frequency distribution and 4, 8, and 16 elements in each ring^13^. The initial geometry parameters, such as radii and the number of elements, are based on a uniformly excited conventional array that has been optimized for minimum side lobe levels (SLLs) within ± 30° scanning range. The second array, A2, has the same geometry as A1 but with a linear frequency distribution^9^. In this array, the frequency offset of elements is calculated using the equation $\delta \omega_{n}^{i} = \delta \omega_{1}^{i} \left( {1 - {{2\varphi_{n}^{i} } \mathord{\left/ {\vphantom {{2\varphi_{n}^{i} } \pi }} \right. \kern-0pt} \pi }} \right)$. In conventional arrays, hexagonal structures are known to offer lower spatial SLLs due to the equal distance between all adjacent elements^25^. Hence, the third array, A3, consists of three hexagonal rings with a linear frequency distribution. In this array, the distance between all adjacent elements is the same, and the total number of elements is calculated by $N_{{}}^{i} = 6i$, while i is the number of rings. Array A4 represents the discular equivalent of A3, maintaining the same frequency distribution and ring configuration. The frequency distribution, rings, and total number of elements in array A5 are similar to the designed discular array (A6), except for the use of a uniform element distribution instead of the nonlinear distribution in A6. Array A6 is the designed discular array, which has undergone the optimization process described earlier.

**Figure 5:**
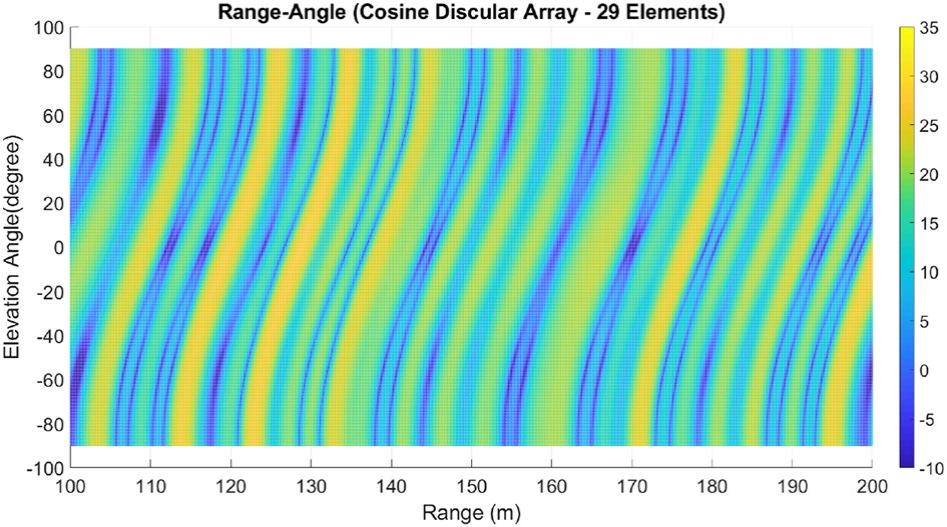
Elevation-range pattern of A1 array.

**Figure 6:**
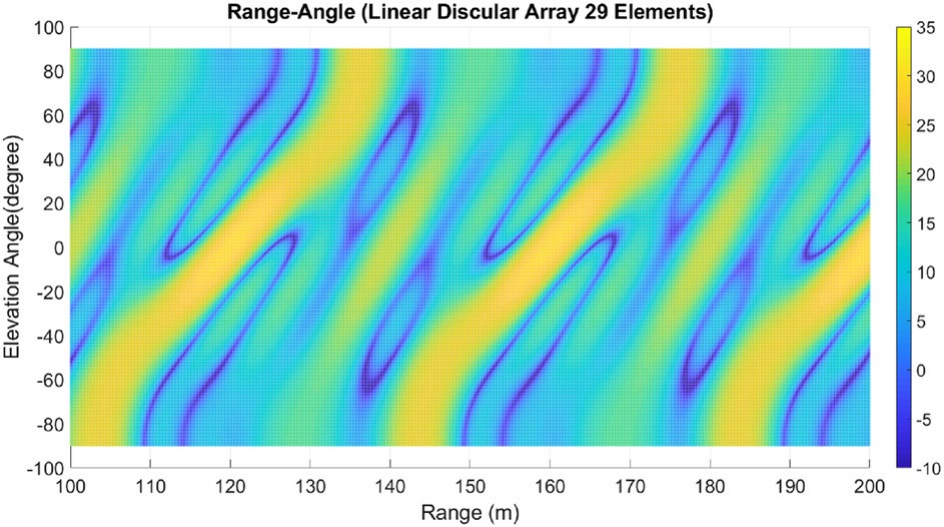
Elevation-range pattern of A2 array.

**Figure 7:**
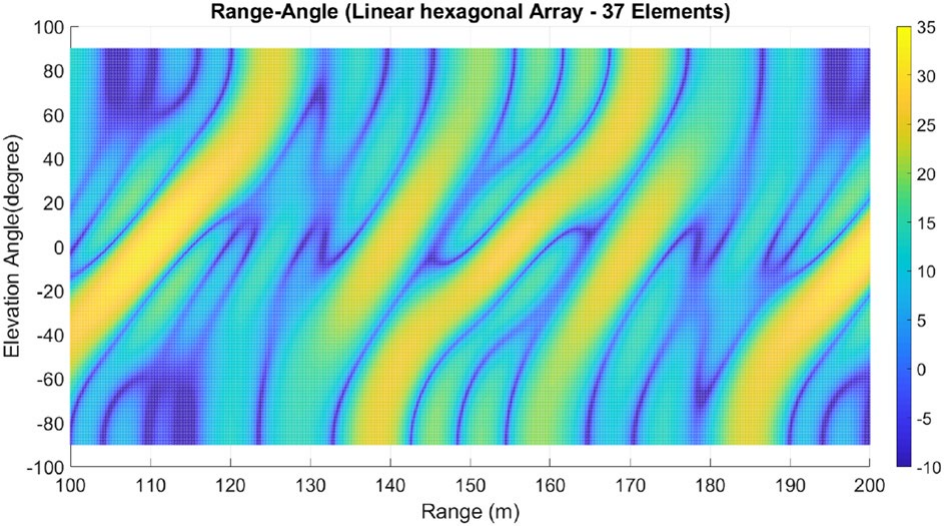
Elevation-range pattern of A3 array.

**Figure 8:**
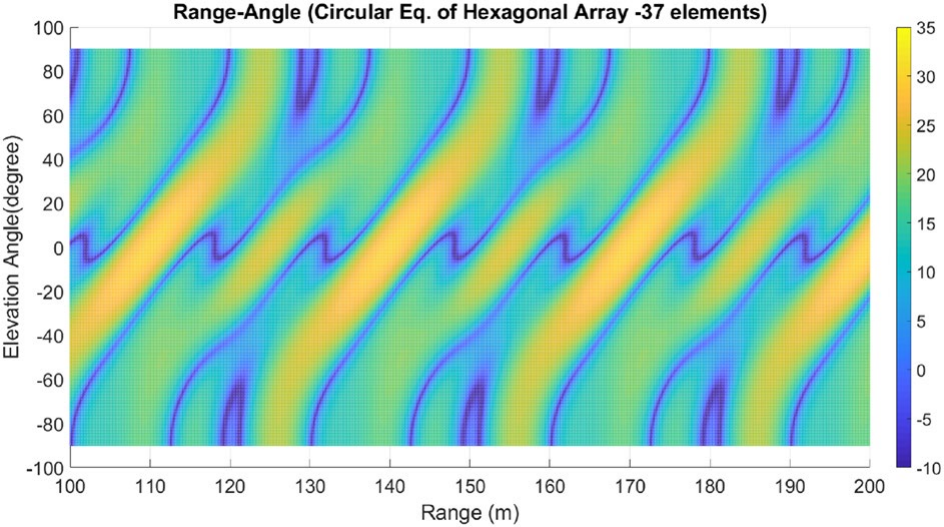
Elevation-range pattern of A4 array.

**Figure 9:**
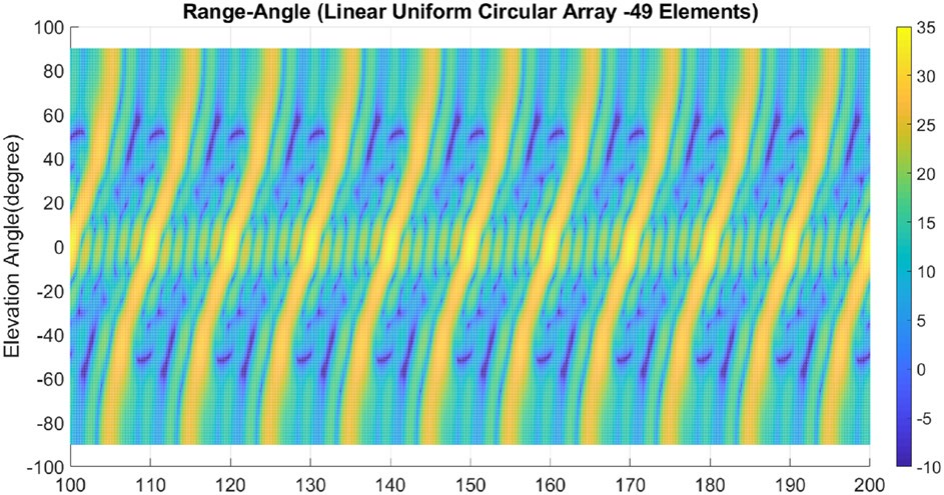
Elevation-range pattern of A5 array.

**Figure 10:**
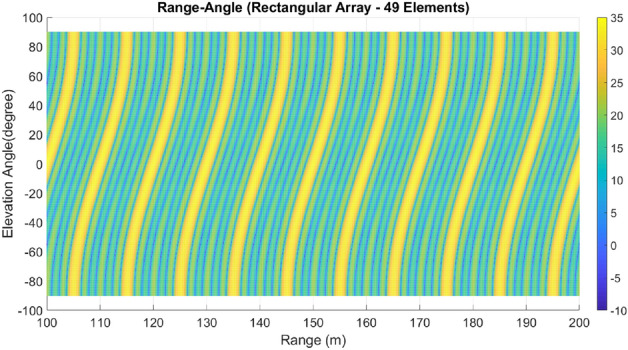
A6 array elevation-range pattern.

**Figure 11:**
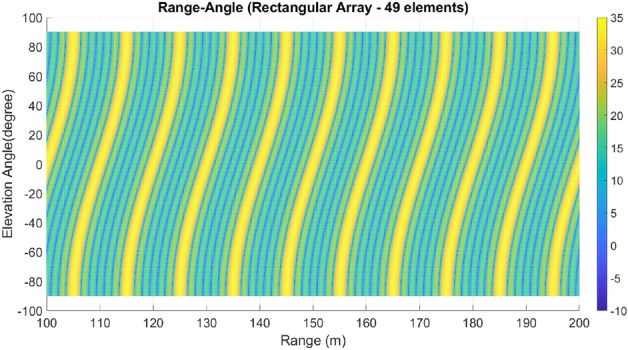
A7 array elevation-range pattern.

Lastly, array A7 represents the optimal rectangular array. Table 1 illustrates the geometrical characteristics of these arrays. Table 2 summarizes and compares the two design parameters, scan rate and angle-changing rate, of these arrays. Additionally, Table 3 describes the Figure of Merit (FoM) of arrays A1 to A7, along with two other arrays from references^9,13^. The range-angle patterns of these arrays are plotted in Figs. 5, 6, 7, 8, 9, 10 and 11 for further analysis and comparison.

**Table 1:** FDA frequency and geometrical distributions.

No	Array geometry (Frequency distribution)	Elements radii in rings in (${\lambda }_{0})$	${L}^{i}$	No. of elements	Frequency distribution
A1	Discular (Uniform)	(0.5), (0.93), (1.35)	4,8,16	28	Cosine
A2	Discular (Uniform)	(0.5), (0.93), (1.35)	4,8,16	28	linear
A3	Hexagonal	(0.5), (0.86,1.0), (1.26,1.5)	6,12,18	36	linear
A4	Discular (Average Hexagonal)	(0.5), (0.93), (1.38)	6,12,18	36	linear
A5	Discular (Uniform)	(0.6), (1.2), (1.75)	8,16,24	49	Uniform
A6	Discular (Non-uniform)	(0.6), (1.2), (1.75)	8,16,24	49	Uniform
A7	Rectangular	(0.5,0.7), (1,1.11,1.41), (1.5,1.58,1.8,2.12)	8,16,24	49	Uniform

**Table 2:** Scan period and angle-changing rate of the simulated arrays.

No	Scan period	Angle-changing rate
A1	$T_{{Scan}} = LCM~\left( {~T_{b} ,~\frac{{T_{b} }}{{\cos \;\pi /8}},\frac{{T_{b} }}{{\cos \;2\pi /8}},\frac{{T_{b} }}{{\cos \;3\pi /8}}} \right)$	$\frac{1}{{\rho }_{1}^{1}cos{\varphi }_{1}^{1} }.\left(\frac{2C{\omega }_{b}}{{\omega }_{0}\mathrm{cos }{\varphi }_{m}.\mathrm{cos}{\theta }_{m}}\right)$
A2	${T}_{Scan}=LCM \left( {T}_{b},{2T}_{b},{4T}_{b},{\frac{4}{3}T}_{b}\right)=4\frac{{T}_{b}}{3}$	$\frac{2}{{0.5}},\frac{2}{{0.93}},\frac{{\sqrt 2 }}{{0.93}},\frac{2}{{1.35}},\frac{{\sqrt 2 }}{{1.35}},\frac{{0.75}}{{1.35.\cos \;\pi /8}},\frac{{0.25}}{{1.35.\cos \;3\pi /8}}\left( {\frac{{2C\omega _{b} }}{{\omega _{0} \cos {\text{~}}\varphi _{m} \cos \theta _{m} }}} \right)$
A3	${T}_{Scan}=LCM ({\frac{9}{7}T}_{b},{\frac{9}{6}T}_{b},{\frac{9}{5}T}_{b},{\frac{9}{4}T}_{b},{\frac{9}{3}T}_{b},{\frac{9}{2}T}_{b},{\frac{9}{1}T}_{b})$	$\frac{1}{ {\rho }_{n}\mathrm{cos}{\varphi }_{n}}\left(\frac{2C{\omega }_{b}}{{\omega }_{0}\mathrm{cos }{\varphi }_{m}\mathrm{cos}{\theta }_{m}}\right), n=1\sim 10$
A4	The same as A3	The same as A3 with different radii
A5	${T}_{Scan}=LCM \left({\frac{1}{3}T}_{b},{\frac{1}{2}T}_{b},{\frac{1}{1}T}_{b}\right)={\frac{1}{3}T}_{b}$	The same as A3 with different radii
A6	The same as A5	The same as A3 with different radii,${\sum }_{i=0}^{M}{\sum }_{n=1}^{{N}_{q}^{i}}(\frac{d\theta }{dt}{)}_{rec}-(\frac{d\theta }{dt}{)}_{n(eq)}=0$
A7	The same as A5	$\frac{1}{ {\rho }_{1}^{1}cos{\varphi }_{1}^{1}}.\left(\frac{2C{\omega }_{b}}{{\omega }_{0}\mathrm{cos }\varphi \mathrm{cos}{\theta }_{m}}\right)$

**Table 3:** Comparing different array performances.

Configuration	N	Gain (dBi)	Normalized SLL in a plane (dBc)			Beamwidth	FoM
			EL-AZ	EL-Time	EL-Range		
A1	29	29.5	− 3.5	− 4.3	− 2.5	15–40	0.34
A2	29	29.25	− 6.2	− 7.2	− 7	20–25	0.69
A3	37	32.5	− 8.2	− 7.5	− 16	24–26	0.97
A4	37	32.5	− 8.1	− 12	− 14	24–26	1.04
A5	49	32.2	− 7.2	− 6	− 7.4	15–20	0.66
**A6**	**49**	**34**	**− 15**	**− 14**	**− 13**	**15–20**	**1.23**
A7	49	34	− 15	− 14	− 12	15–20	1.2
Discular^9^	37	–	–	–	− 5.2	30	–
Discular^13^	37	–	–	− 6 dB	–	–	

Significant values are in bold.

## Comparison of the array’s performances

### Arrays comparison

While it is essential to compare the performance of the simulated arrays across all three defined planes, the range-angle pattern can serve as a comprehensive representation, allowing for the simultaneous visualization of both temporal and spatial sidelobes of the array. Furthermore, Table 3 includes the evaluation of the figure of merit (FoM), which incorporates the impact of elevation-azimuth and elevation-time patterns on the overall performance of the array.

#### Elevation-range pattern comparison

In the designed array, the reference element exhibits a frequency offset of 30 MHz from the center element. As a result, the fundamental scan period of the array is calculated to be 33.3 ns. Considering the free space wave velocity, we can estimate that the wave travels approximately 10 m during the fundamental scan period. Consequently, within a range of 100 m, we anticipate the presence of 10 distinct pattern periods. Upon comparing Figs. 5, 6, 7, 8, 9, 10 and 11, it becomes apparent that only in A5, A6, and A7 are all ten periods visible in the pattern. On the other hand, the range-angle patterns of A2, A3, and A4 exhibit 2.5, 2, and 3.5 periods, respectively. The side lobes of the range-angle pattern are measured from the areas of high gain (highlighted in yellow) located between the distinct patterns. In these arrays, the side lobes are considerably higher compared to A6 and A7. No discernible periodic pattern can be observed in the range-angle pattern of array A1. From this analysis, we can conclude that by linearizing the frequency distribution in arrays A5, A6, and A7, the periodicity and stability of the pattern across multiple scan rounds are enhanced.

#### FoM comparison

The data presented in Table 3 highlights that the arrays (A6-A7), (A6-A7), and A3 exhibit the lowest Side Lobe Levels (SLLs) in the Elevation-Azimuth, Elevation-Time, and Elevation-Range planes, respectively. A6 and A7 are the designed discular and rectangular arrays, where the time and spatial characteristics of the Array Factor (AF) are controlled through the design process. On the other hand, the results from array A3 affirm the advantage of the hexagonal geometry in achieving lower SLLs in the Elevation-Range plane. An important observation is that the Figure of Merit (FoM) A6 surpasses that of the A7 due to its lower SLL in the elevation-range plane. It is worth considering that removing the edges of the arrays could potentially enhance the spatial purity of the pattern.

#### Beamwidth comparison

Among all the simulated arrays, A5, A6, and A7 exhibit the narrowest beamwidths within the scanning angle range of ± 30°. This characteristic can be attributed to the higher gain and larger number of elements present in these arrays. On the other hand, A3 and A4 demonstrate minimal variations in beamwidth within the same scanning angle range. This finding further supports the advantage of hexagonal geometries in achieving desirable patterns at end-fire angles. Despite the favorable performance of hexagonal configurations in the elevation-azimuth plane, their FoM is lower compared to the designed arrays. This discrepancy arises from the higher Side Lobe Levels (SLL) observed in the elevation-time plane, indicating a lack of control over the time behavior of arrays A3 and A4.

#### Some other points

**Figure 12:**
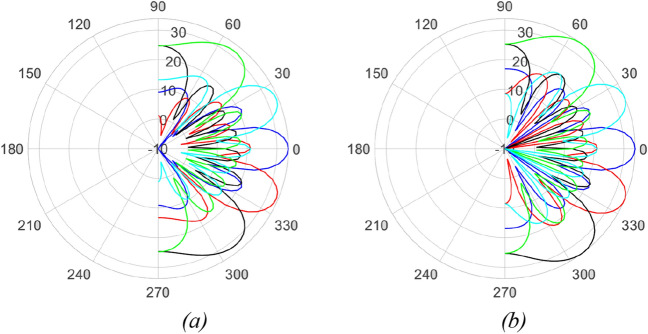
Cumulative elevation pattern of the designed (**a**) rectangular and (**b**) discular array over time (dB).

**Figure 13:**
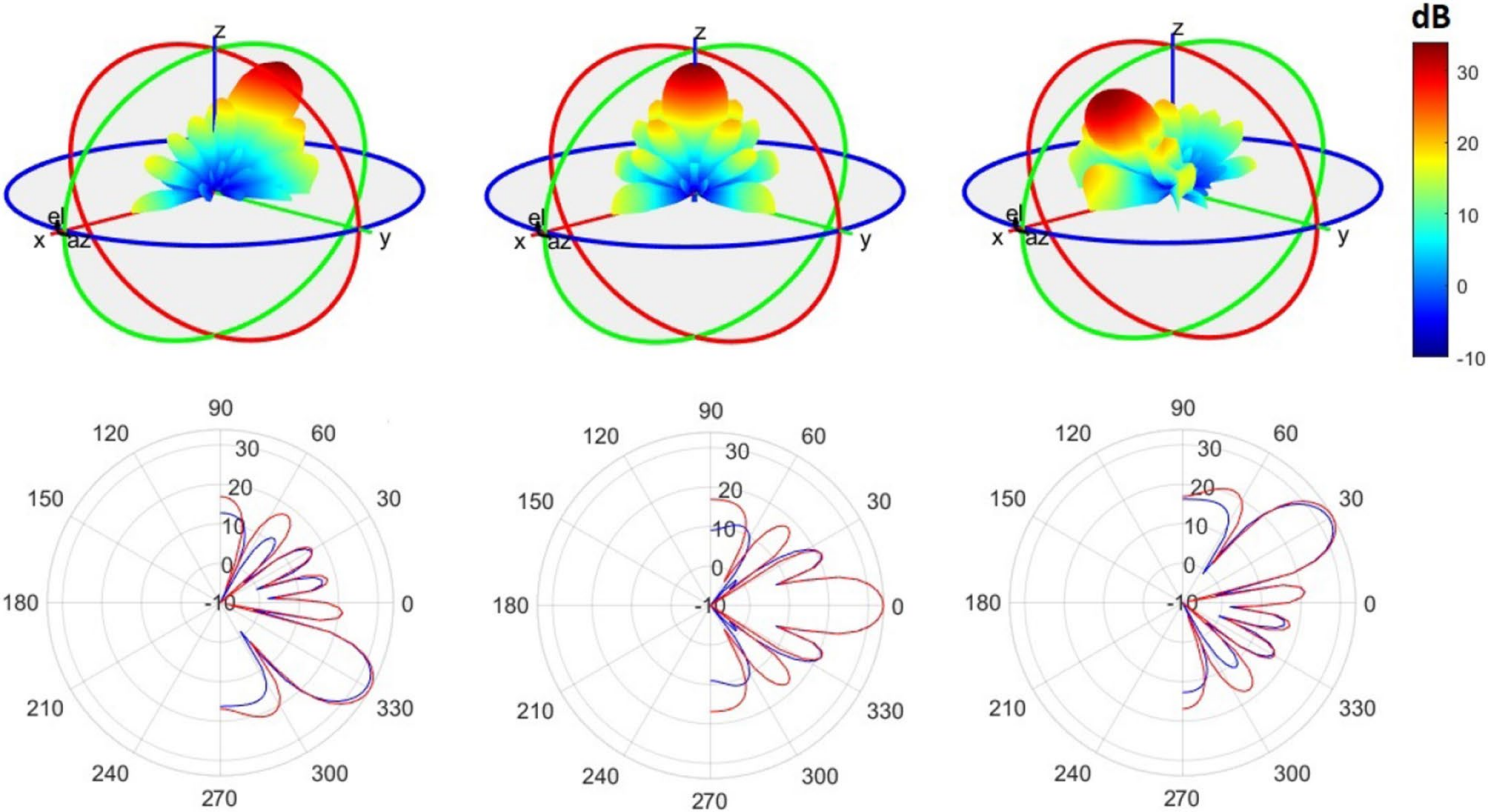
First row: 3D pattern of the designed circular array in elevation angles of − 30, 0, 30, Second row: comparing elevation pattern of the designed rectangular (blue line) and equivalent circular array (red line).

- Arrays A5 and A6 are nearly identical, with the only distinction being the spatial distribution of elements in the second and third rings. In A5, the elements are uniformly distributed, while A6 utilizes a shifted cosine function form. The lower FoM of A5 (0.66) compared to A6 (1.23) highlights the significant impact of geometry on both the temporal and spatial behavior of the pattern.
- The last two rows of Table 3 summarize the results of the arrays mentioned in references^9,13^. Although these arrays exhibit higher SLLs than the designed array in a single plane, their FoM could not be defined due to a lack of pattern performance in all three defined planes.
- Considering the lower SLLs and higher FoM of A6 compared to the other arrays, it can be concluded that the designed array outperforms the other simulated arrays. To further investigate the time behavior of A6, the elevation-time and elevation-azimuth patterns of the array are plotted in Figs. 12 and 13.
- Fig. 12 illustrates the cumulative elevation-time patterns of arrays A6 and A7, showcasing the repetitive pattern in multiple scanning rounds. Both arrays demonstrate a consistent angle without any shift in each of the ten scanning rounds.
- In Fig. 13, the elevation-time patterns of A6 and A7 are compared at three different angles. The first row of the figure presents the 3D pattern of array A6. The bottom row depicts that A6 exhibits higher SLLs near the end-fire region compared to the optimal array A7. However, overall, the maximum SLL for both arrays remains the same, as indicated in Table 3.
  $$FoM= \sum \frac{{-SLL}_{dB}}{Gain}$$

### Space-frequency diagram of FDA

**Figure 14:**
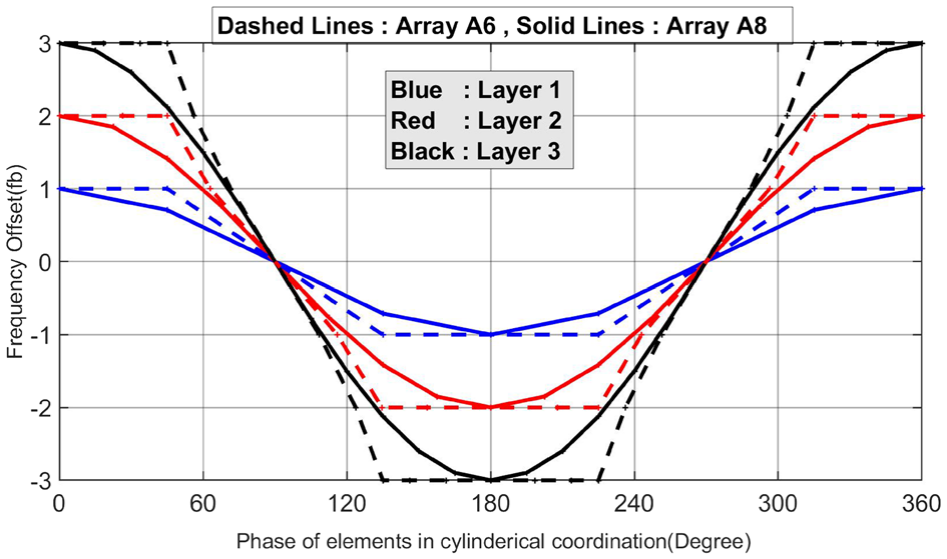
Space-frequency diagram of the arrays A6 and A8.

To visualize the main design parameters of the array in a single plot, a space-frequency diagram is generated for discular Frequency Diverse Arrays (FDAs), as shown in Fig. 14. This plot combines the frequency and phase of the cylindrical coordinates of the array elements. The horizontal axis represents the phase, while the vertical axis represents the frequency offset of the elements from the center element. The blue, red, and black lines represent the spatial distributions of elements in the first, second, and third rings of the arrays, respectively.

Figure 14 compares the space-frequency diagram of array A6 (dashed lines) with a reference discular array A8 (solid lines). The reference array shares the same number of rings, total number of elements, and radii as array A6. However, the frequency distribution in the reference array follows a cosine form (to maintain a unique angle-changing rate), and the elements are uniformly arranged on the rings. In array A6, the element density is higher around $\varphi ={45}^{^\circ }$ compared to the x- and y-axis. Furthermore, the frequency offsets of elements in array A6 tend to converge to the nearest integer value of the frequency offset of the corresponding element in array A8. The designed reference array exhibits a very similar pattern and the same FoM as array A1. It is noteworthy that arrays A1 and A6 have the lowest and highest FoM among the simulated arrays, respectively. It can be inferred that to transform array A8 into array A6, the elements should be moved in a way that minimizes the surface area between these two space-frequency diagrams, taking into account the need for quantized values for the frequency offsets. This transformation converts the cosine frequency distribution in array A8 to the shifted cosine element distribution in array A6. On the other hand, to achieve an optimal FDA, further research is required to develop a design approach based on the space-frequency diagram, focusing on minimizing the surrounding area between these two curves, considering their sign. Table 4 provides a comparison of the geometry parameters and simulation results for arrays A8 (with the worst performance) and A6 (with the best performance).

**Table 4:** Comparing array configurations and performances of the best and the worst array.

	A6	A8
Array geometry	Discular	Discular
Total number of elements	49	49
Number of rings	3	3
Number of elements in each ring	8, 16, 24	8, 16, 24
Distribution of the elements in the rings	Cosine	Linear
Frequency distribution	Linear	Cosine
Angle-changing rate	Equation 22 (Sum of the error function is zero)	Unique
Scan rate	${T}_{Scan}=LCM \left({\frac{1}{3}T}_{b},{\frac{1}{2}T}_{b},{\frac{1}{1}T}_{b}\right)={\frac{1}{3}T}_{b}$	$\frac{1}{{\rho }_{1}^{1}cos{\varphi }_{1}^{1} }.\left(\frac{2C{\omega }_{b}}{{\omega }_{0}\mathrm{cos }{\varphi }_{m}.\mathrm{cos}{\theta }_{m}}\right)$
FoM	1.23	0.65

## Conclusion

**Figure 15:**
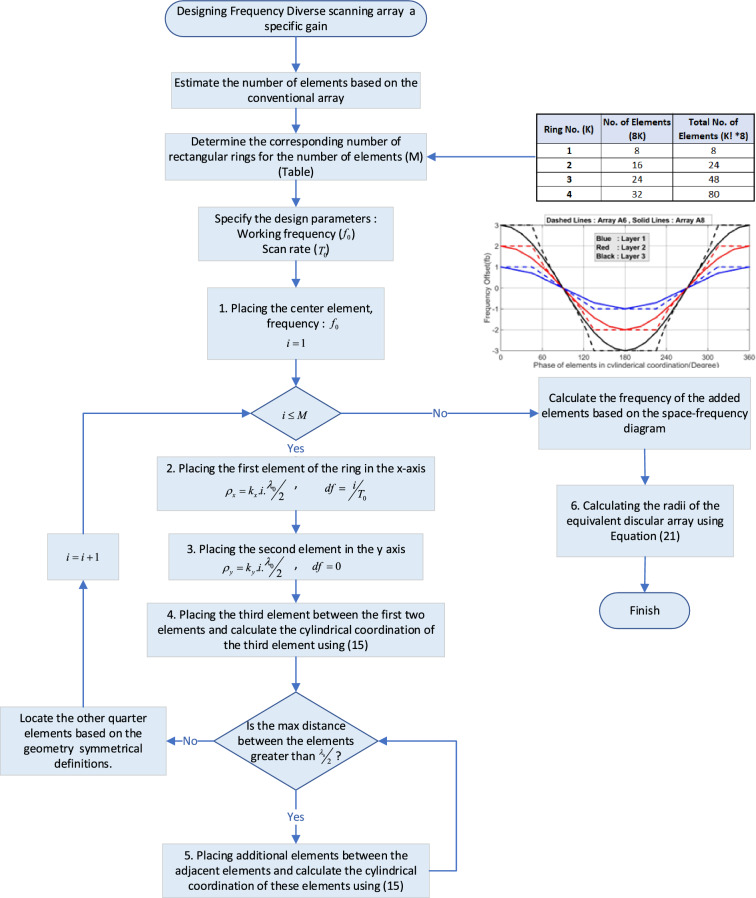
Discular array designing flowchart.

In this research, a straightforward formulation is derived by defining symmetric criteria for the frequency and spatial configuration of frequency-diverse arrays (FDAs). This formulation is used to extract and analyze two important design parameters of the array. Based on the analysis results, a novel algorithm is proposed to design the frequency and spatial distribution of a planar FDA for a one-dimensional scanning pattern. To evaluate the performance of the designed array, multiple commonly used FDA geometries have been redesigned, and their patterns are compared with those of the designed array in the three defined planes. To facilitate a better comparison between FDAs, a space-frequency diagram is introduced, allowing for a comprehensive analysis of multiple FDA geometries in a single diagram. By comparing the space-frequency diagrams of arrays with the best and worst pattern performances, significant insights about the FDA geometries are revealed. These findings can serve as a basis for further research to develop more advanced algorithms for designing two-dimensional scanning arrays. To simplify the design process, a flowchart of the design algorithm is prepared in Fig. 15, summarizing the design steps. The suggested algorithm aims to find the optimum geometry for a planar FDA capable of one-dimensional scanning, although it can be generalized to other scanning patterns. The derived formulation provides a straightforward approach to analyzing both scanning and steering patterns. Furthermore, the algorithm can be modified to obtain a time-invariant FDA for steering applications.

## Supplementary Information

Supplementary Information.

## Data Availability

The manuscript is not involved with any clinical data, although all simulated and theoretical data would be available by email request to the corresponding author.
