# Supplementary material for: Joint frequency space design approach for efficient planar frequency diverse arrays
Source: Sci Rep. 2023 Jul 23;13:11894. doi: 10.1038/s41598-023-39024-6 (PMC10363549; doi:10.1038/s41598-023-39024-6)
Supplement: Supplementary file 1 — Supplementary Information. [file 41598_2023_39024_MOESM1_ESM.docx]

**Appendix A**

**Symmetric Concentric Ring Frequency Diverse Array**

For the *M* ring array, each ring *i* has *N^i^* elements with the central element being *i* = 0, has the array factor

(A1)

where $R_{0}$is the position vector of the observation point and $r_{n}^{i}$is the *n*^th^ element position in the *i*^th^ ring. In the far-field, Equation (A1) can be written as

(A2)

Using and$\omega_{n}^{i}=\omega_{c}+\delta\omega_{n}^{i}$with $\delta\omega_{n}^{i}\ll\omega_{c}$, and

Then

The AF after normalization to $\exp(j\omega_{c}(t-\frac{R_{0}}{c})/R_{0}$ becomes

(A3)

For conventional arrays, in which the frequency-dependent term turns to zero, the AF can be expressed in other forms [6], [24]; by defining $\tau=t-\frac{R_{0}}{c}$and$\gamma_{n}^{i}(\theta)=\omega_{n}^{i}\frac{\rho^{i}}{c}\sin\theta$, the AF becomes:

(A4)

The first term represents spatial behavior, and the second describes the time behavior of the array. In this formulation, the number of terms equals the number of the array elements. We tried to define some symmetry rules to reduce the number of independent terms. If we choose an even number for each ring and place the elements uniformly on the ring, the placement will have symmetry along the x- and y-axis.

(A5)

where determines the location of the first element in the ring *i* of the array. Here, we have four possibilities. First, , the first element of ring *i* is located on the x-axis, and no elements on the y-axis. Second, and elements on the y-axis. Third, and elements on the y-axis. Fourth, and no elements on the y-axis. To relate spatially symmetrical elements, the frequencies of these elements are also considered symmetrically. Thus, the number of elements on each ring is even except for the center element. Thus, the number of individual terms is reduced to elements in the first quarter, including those on the axes. If we define the frequencies of the elements to be evenly symmetrical with the x-axis and oddly symmetrical with the y-axis, then half of the number of each ring *i* is $N_{h}^{i}=N^{i}/2$, we have

$\delta\omega_{n}^{i}=\left\{\begin{matrix} -\delta\omega_{N_{h}^{i}-n+j}^{i} n\leq N_{h}^{i} \\ \delta\omega_{N^{i}-n+j}^{i} n>N_{h}^{i} \end{matrix}\right. when j=\left\{\begin{matrix} 2 elements on x-axis \\ 1 no elements on x-axis \end{matrix}\right.$ (A6a)

$A_{n}^{i}=\left\{\begin{matrix} A_{N_{h}^{i}-n+j}^{i} n\leq N_{h}^{i} \\ A_{N^{i}-n+j}^{i} n>N_{h}^{i} \end{matrix}\right. when j=\left\{\begin{matrix} 2 elements on x-axis \\ 1 no elements on x-axis \end{matrix}\right.$ (A6b)

Where

$$m_{\left\{\begin{matrix} x \\ y \end{matrix}\right\}}^{i}=\left\{\begin{matrix} 0 no elements on \left\{\begin{matrix} x \\ y \end{matrix}\right\}-axis \\ 1 elements on \left\{\begin{matrix} x \\ y \end{matrix}\right\}-axis \end{matrix}\right.$$

Fig. 1a illustrates the elements’ locations of the first quarter of the proposed discular planar FDA. In this configuration, elements are distributed symmetrically around the x- and y-axis. Once the frequency and location of elements located in the first quarter are designed, other elements' frequency and location can be calculated. The defined symmetrical conditions were applied in many studies like [7]-[26]. Linear or sinusoidal frequency distribution also satisfies these symmetrical rules. Equations (A7) and (A8) describe the proposed linear and cosine distributions.

(Linear) (A7)

(Cosine) (A8)

In which $\omega_{b}$ is the fundamental beat frequency of the array. Notice that the elements on the y-axis have $\delta\omega_{n}^{i}=0$. The adjusting factor to generalize the AF expression is for elements (not on the x- or y-axis or only on one of them, CF= 0, but with elements on both x- and y-axis CF= 1, Collecting the upper half of the elements plus those on the axes:

Using and

Using (6a) and the odd characteristic of the offset frequencies, then

(A9)

and for the remaining elements located below the x-axis, we have:

Using

(A10)

By adding the terms that have the same frequency offsets in (A9) and (A10) and using sinusoidal simplifications:

However, for a compact form

(A11a)

(A11b)

where $N_{q}^{i}=N_{h}^{i}/2=N^{i}/4$ and$a_{n}=2$ for and and $a_{n}=4$for other.

Using these identities:

$$\begin{matrix} \sin(a\pm b)=\sin(a)\cos(b)\pm\cos(a)\sin(b) \\ \cos(a\pm b)=\cos(a)\cos(b)\mp\sin(a)\sin(b) \\ \sin(a)\cos(b)=\frac{1}{2}(\sin(a+b)+\sin(a-b)) \\ \cos(a)\sin(b)=\frac{1}{2}(\sin(a+b)-\sin(a-b)) \\ \cos(a)\cos(b)=\frac{1}{2}(\cos(a+b)+\cos(a-b)) \\ \sin(a)\sin(b)=\frac{1}{2}(-\cos(a+b)+\cos(a-b)) \end{matrix}$$

(A12)

(A13)

The condition applied in the elements' amplitudes is defined in (A6b). In this approach, locating the elements in the first quarter of the plane helps determine the other element's location and frequency offsets. For the planar array of Fig. 1a with 29 elements (three rings plus the center element), the number of separate terms in the AF is reduced to 11, which is much easier for design and optimization approaches.

For conventional array if$\delta\omega_{n}^{i}=0$ , the formula converges to:

Forcing the symmetry limits the number of independent amplitudes and frequencies that should be optimized. Although to have symmetrical characteristics in space, one should select a configuration with such symmetry. Even this formulation can simplify AF relation in the conventional arrays, but the advantages are more significant in FDA. In the next section, using this formulation, the scan period and angle-changing rate of arrays are calculated, and several arrays with different scan rates are also designed.

In the previous section, the AF of the discular array was calculated. The same approach can be followed to form AF of any general configuration. Figs. 1b and 1c show three-layer hexagonal and rectangular lattices and their equivalent circular arrays. The circles' radii in the equivalent array are the average radius of all circles representing the layer.

## B. Pattern Analysis

In conventional arrays, sidelobes and beamwidth are usually used to compare different array performances. In this study, two other time-related parameters are added, scan rate and angle-changing rate. These two later parameters are responsible for the pattern fluctuation over time and SLLs. The second cosine function in (A11b) is responsible for the time variation of the pattern.

The location of the maximum steering angle because of the $n^{th}$ element of the $i^{th}$ layer can be calculated from:

Where is the desired beam direction. Then, the time that the $n^{th}$ element the $i^{th}$ring will have its maximum should be at

By averaging for all array factor terms, the location of the steering angle in time and range is obtained. We should also state something about the range

$$t_{\max}|_{n}^{i}=\tau_{\max}|_{n}^{i}+\frac{R_{0}}{c} or R_{0}=c(t_{\max}|_{n}^{i}-\tau_{\max}|_{n}^{i})$$

**Appendix B**

**Optimum radii of equivalent discular array**

For the rectangular array, the value of the angle changing rate is equal for all the AF terms, which is calculated form

(B1)

This value is different for every $\theta_{\max}$ and . However, in (B1), all terms of the AF $\theta_{\max}$ and is the same if all $\left.ACR\right|_{n}^{i}$ in (B2) converge to a single value, as the rectangular array.

(B2)

To design a discular array, a cost function has been defined to calculate the radius of the rings. The cost function is defined as the summation of the difference between the ACR (the factor of angle changing rate in a rectangular array) and the values for each array factor term in the discular array.

$$\sum_{i=0}^{M} \sum_{n=1}^{N_{q}^{i}} \left. \frac{d\theta}{dt} \right|_{rec}-\left. \frac{d\theta}{dt} \right|_{n(eq)}=K\sum_{i=0}^{M} \sum_{n=1}^{N_{q}^{i}} \left( \frac{{\delta\omega}_{1}^{i}}{\rho_{1}^{i}}-\frac{\delta\omega_{n}^{i}}{\rho_{eq}^{i}\cos\varphi_{n}^{i}} \right)=0$$

(B3)

The $\left.ACR\right|_{n}^{i}$for all terms in the rectangular array is equal to

(B4)

Therefore Equation (B3) for each ring can be simplified to

$N_{q}^{i}.\frac{{\delta\omega}_{1}^{i}}{\rho_{1}^{i}}- \sum_{n=1}^{N_{q}^{i}} \left( \frac{\delta\omega_{n}^{i}}{\rho_{eq}^{i}\cos\varphi_{n}^{i}} \right)=0$ (B5)

considering the frequency offset of the rectangular and discular is chosen to be equal and the $\rho_{eq}^{i}$ is the same for all elements in the ring, the (B5) is simplified to

$$\frac{1}{\rho_{eq}^{i}}.\sum_{n=1}^{N_{q}^{i}} \left( \frac{1}{\cos\varphi_{n}^{i}} \right)=N_{q}^{i}.\frac{1}{\rho_{1}^{i}}$$

$\rho_{eq}^{i}= \frac{1}{N_{q}^{i}}.\sum_{n=1}^{N_{q}^{i}} \rho_{1}^{i}\left( \csc\varphi_{n}^{i} \right)=\frac{1}{N_{q}^{i}}.\sum_{n=1}^{N_{q}^{i}} \rho_{n}^{i}$ (B6)

Equation (B6) describes the average radius of all elements in the layer. By some simplification, we can conclude that the summation of radii changes in the discular array compared to the rectangular array converges to zero.

$$N_{q}^{i}\rho_{eq}^{i}-\sum_{n=1}^{N_{q}^{i}} \rho_{n}^{i}=0= \sum_{n=1}^{N_{q}^{i}} \left( \rho_{n}^{i}-\rho_{eq}^{i} \right)$$
